# The genome sequence of the Beautiful China-mark moth *Nymphula nitidulata *(Hufnagel, 1767)

**DOI:** 10.12688/wellcomeopenres.21102.1

**Published:** 2024-03-08

**Authors:** Douglas Boyes, Peter O. Mulhair, University of Oxford and Wytham Woods Genome Acquisition Lab, Darwin Tree of Life Barcoding collective, Wellcome Sanger Institute Tree of Life Management, Samples and Laboratory team, Wellcome Sanger Institute Scientific Operations: Sequencing Operations, Wellcome Sanger Institute Tree of Life Core Informatics team, Tree of Life Core Informatics collective, Darwin Tree of Life Consortium

**Affiliations:** 1 UK Centre for Ecology & Hydrology, Wallingford, England, UK; 2 University of Oxford, Oxford, England, UK

**Keywords:** Nymphula nitidulata, Beautiful China-mark moth, genome sequence, chromosomal, Lepidoptera

## Abstract

We present a genome assembly from an individual female *Nymphula nitidulata* (the Beautiful China-mark moth; Arthropoda; Insecta; Lepidoptera; Crambidae). The genome sequence is 635.8 megabases in span. Most of the assembly is scaffolded into 31 chromosomal pseudomolecules, including the Z and W sex chromosomes. The mitochondrial genome has also been assembled and is 15.36 kilobases in length. Gene annotation of this assembly on Ensembl identified 20,031 protein coding genes.

## Species taxonomy

Eukaryota; Opisthokonta; Metazoa; Eumetazoa; Bilateria; Protostomia; Ecdysozoa; Panarthropoda; Arthropoda; Mandibulata; Pancrustacea; Hexapoda; Insecta; Dicondylia; Pterygota; Neoptera; Endopterygota; Amphiesmenoptera; Lepidoptera; Glossata; Neolepidoptera; Heteroneura; Ditrysia; Obtectomera; Pyraloidea; Crambidae; Nymphulinae; *Nymphula*; *Nymphula nitidulata* (Hufnagel, 1767) (NCBI:txid1594316).

## Background

*Nymphula nitidulata*, aptly named the Beautiful China-mark moth, is one of the more distinctive and charismatic species of the subfamily Acentropinae. The forewings are a shining white with brown, rounded markings. Like other species in the subfamily, *N. nitidulata* is associated with freshwater environments, where the larvae live, while the adults are terrestrial ( De-Freitas *et al.*, 2019; Pabis, 2018). It is a small moth with a wingspan of 20–25 mm. Fairly widespread throughout Britain and Ireland, this species is classified as local by the Butterfly Conservations’ Microlepidoptera report. This species comes to light and is easily disturbed by day from vegetation near the waterside.

The larvae are a bright yellow with a dark brown dorsal line and pale brown head. They live in streams, lakes, as well as fens and marshes and feed on bur-reed ( *Sparganium* spp.) and yellow water-lily ( *Nuphar lutea*). The genome of this species is a key addition to the underrepresented aquatic insects ( Hotaling *et al.*, 2020), and will provide insights into how this subfamily adapted to live in freshwater habitats.

The genome of the Beautiful China-mark, *Nymphula nitidulata*, was sequenced as part of the Darwin Tree of Life Project, a collaborative effort to sequence all named eukaryotic species in the Atlantic Archipelago of Britain and Ireland. Here we present a chromosomally complete genome sequence for *Nymphula nitidulata*, based on one female specimen from Wytham Woods.

## Genome sequence report

**Figure 1.**
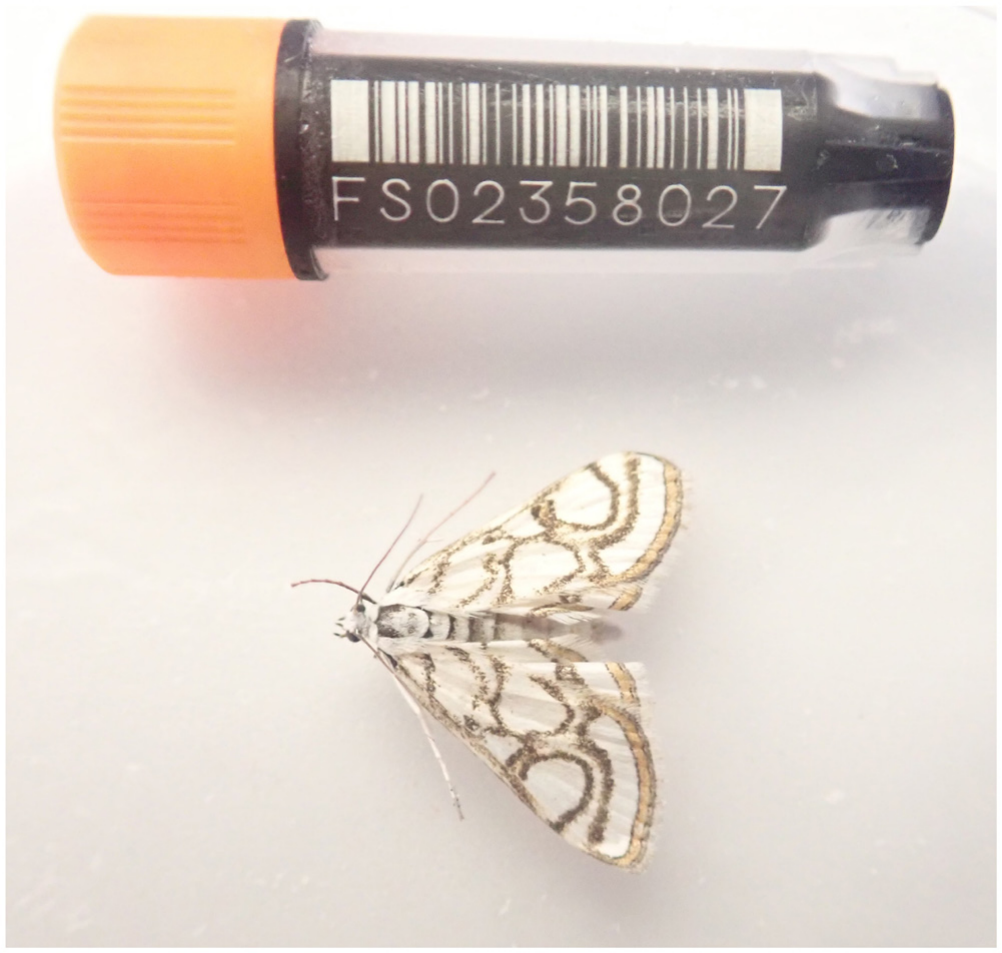
Photograph of the *Nymphula nitidulata* (ilNymNiti1) specimen used for genome sequencing.

The genome was sequenced from one female *Nymphula nitidulata* ( Figure 1) collected from Wytham Woods, Oxfordshire, UK (51.77, –1.33). A total of 41-fold coverage in Pacific Biosciences single-molecule HiFi long reads was generated. Primary assembly contigs were scaffolded with chromosome conformation Hi-C data. Manual assembly curation corrected 6 missing joins or mis-joins and removed one haplotypic duplications, reducing the assembly length by 0.30% and the scaffold number by 4.65%, and increasing the scaffold N50 by 3.51%.

**Figure 2.**
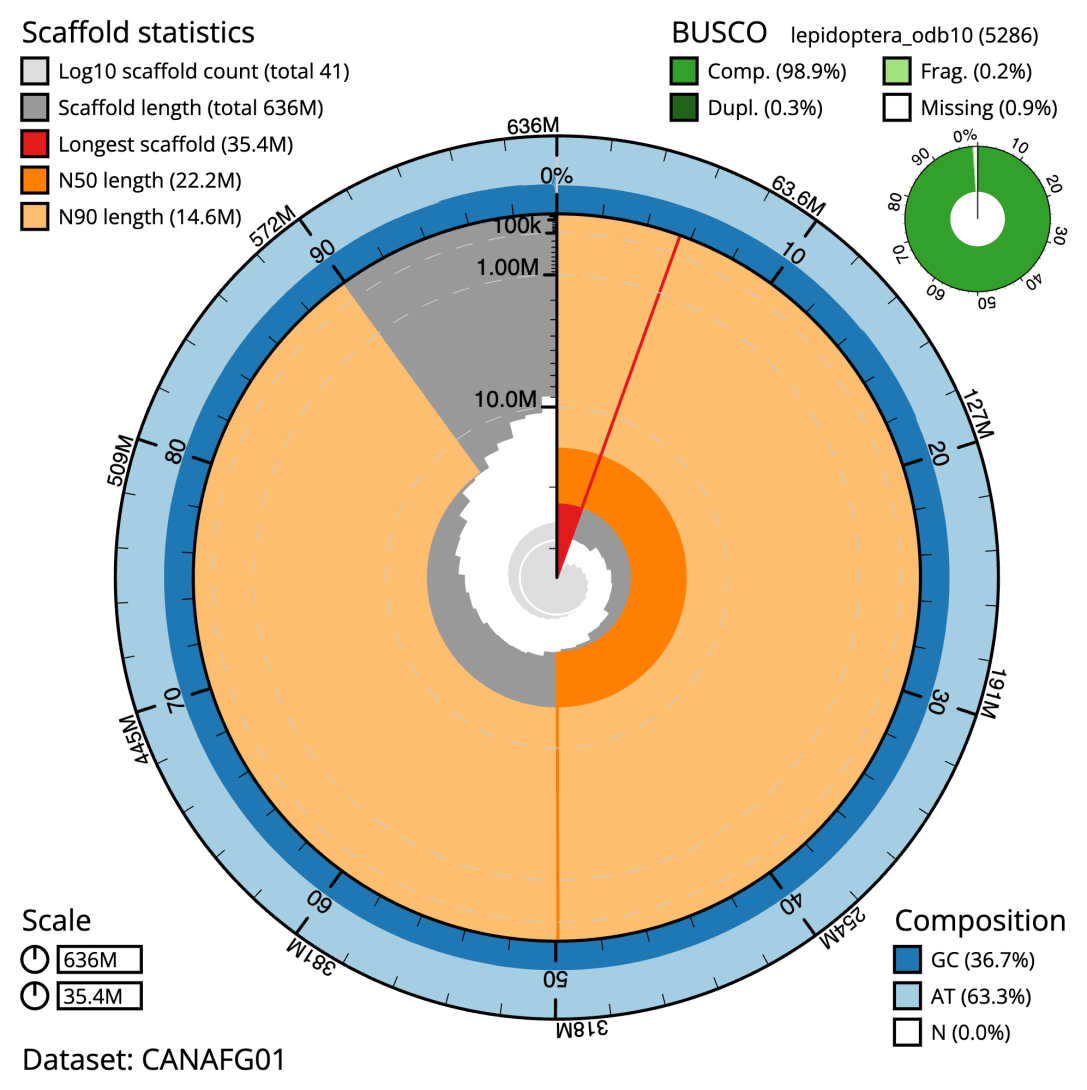
Genome assembly of *Nymphula nitidulata*, ilNymNiti1.1: metrics. The BlobToolKit snail plot shows N50 metrics and BUSCO gene completeness. The main plot is divided into 1,000 size-ordered bins around the circumference with each bin representing 0.1% of the 635,785,502 bp assembly. The distribution of scaffold lengths is shown in dark grey with the plot radius scaled to the longest scaffold present in the assembly (35,422,410 bp, shown in red). Orange and pale-orange arcs show the N50 and N90 scaffold lengths (22,210,000 and 14,630,000 bp), respectively. The pale grey spiral shows the cumulative scaffold count on a log scale with white scale lines showing successive orders of magnitude. The blue and pale-blue area around the outside of the plot shows the distribution of GC, AT and N percentages in the same bins as the inner plot. A summary of complete, fragmented, duplicated and missing BUSCO genes in the lepidoptera_odb10 set is shown in the top right. An interactive version of this figure is available at https://blobtoolkit.genomehubs.org/view/CANAFG01/dataset/CANAFG01/snail.

**Figure 3.**
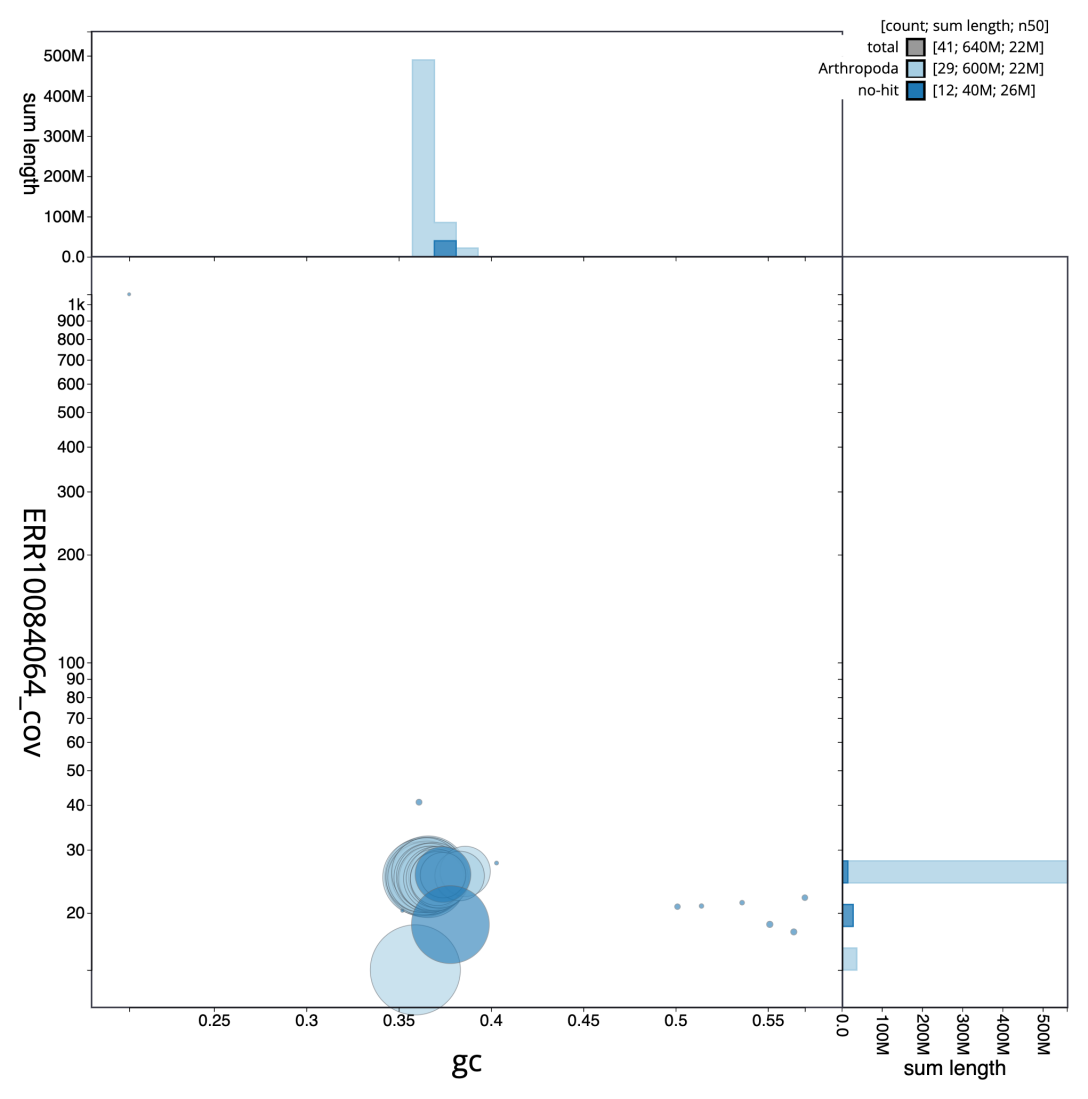
Genome assembly of *Nymphula nitidulata*, ilNymNiti1.1: BlobToolKit GC-coverage plot. Scaffolds are coloured by phylum. Circles are sized in proportion to scaffold length. Histograms show the distribution of scaffold length sum along each axis. An interactive version of this figure is available at https://blobtoolkit.genomehubs.org/view/CANAFG01/dataset/CANAFG01/blob.

**Figure 4.**
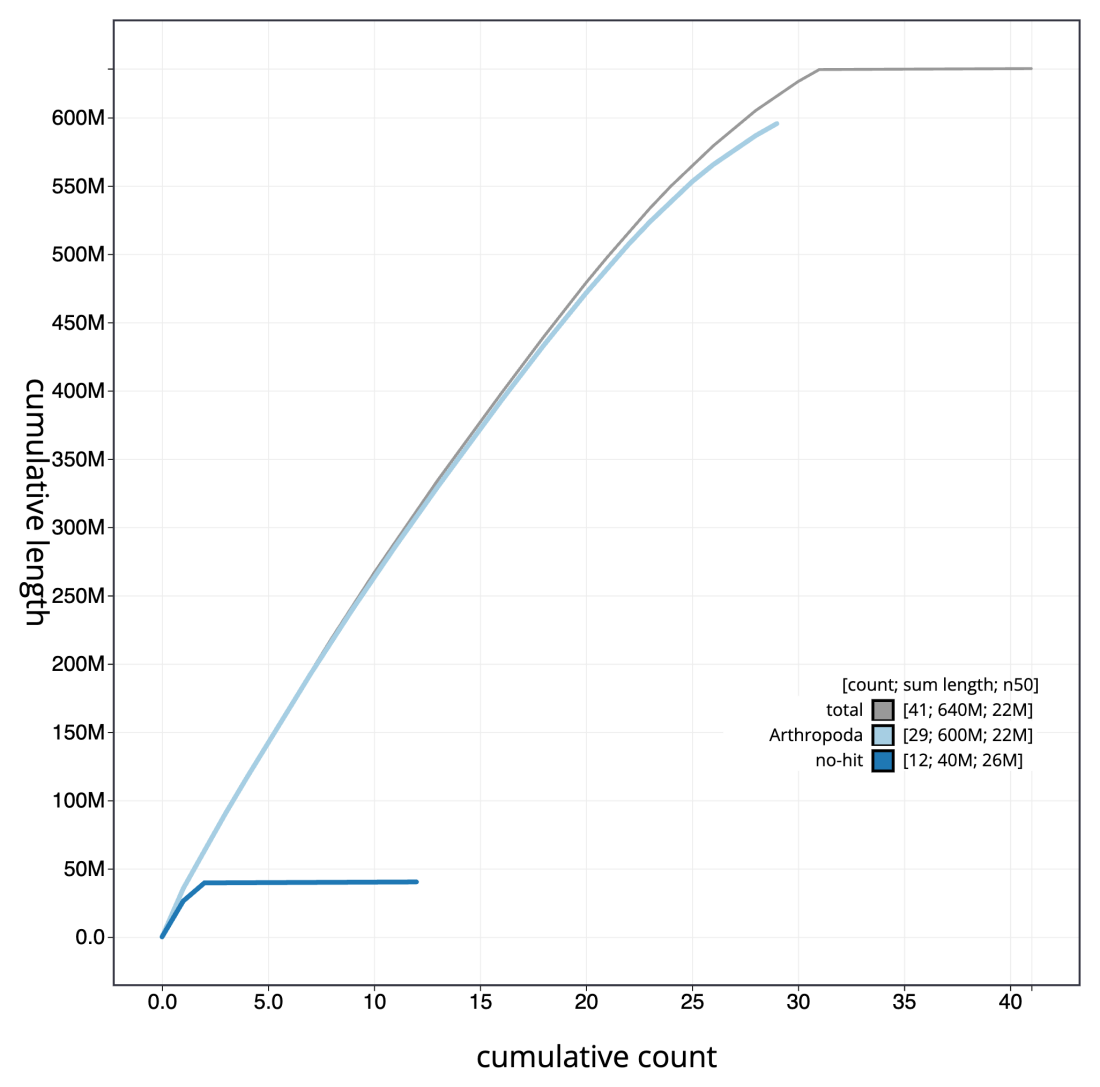
Genome assembly of *Nymphula nitidulata*, ilNymNiti1.1: BlobToolKit cumulative sequence plot. The grey line shows cumulative length for all scaffolds. Coloured lines show cumulative lengths of scaffolds assigned to each phylum using the buscogenes taxrule. An interactive version of this figure is available at https://blobtoolkit.genomehubs.org/view/CANAFG01/dataset/CANAFG01/cumulative.

**Figure 5.**
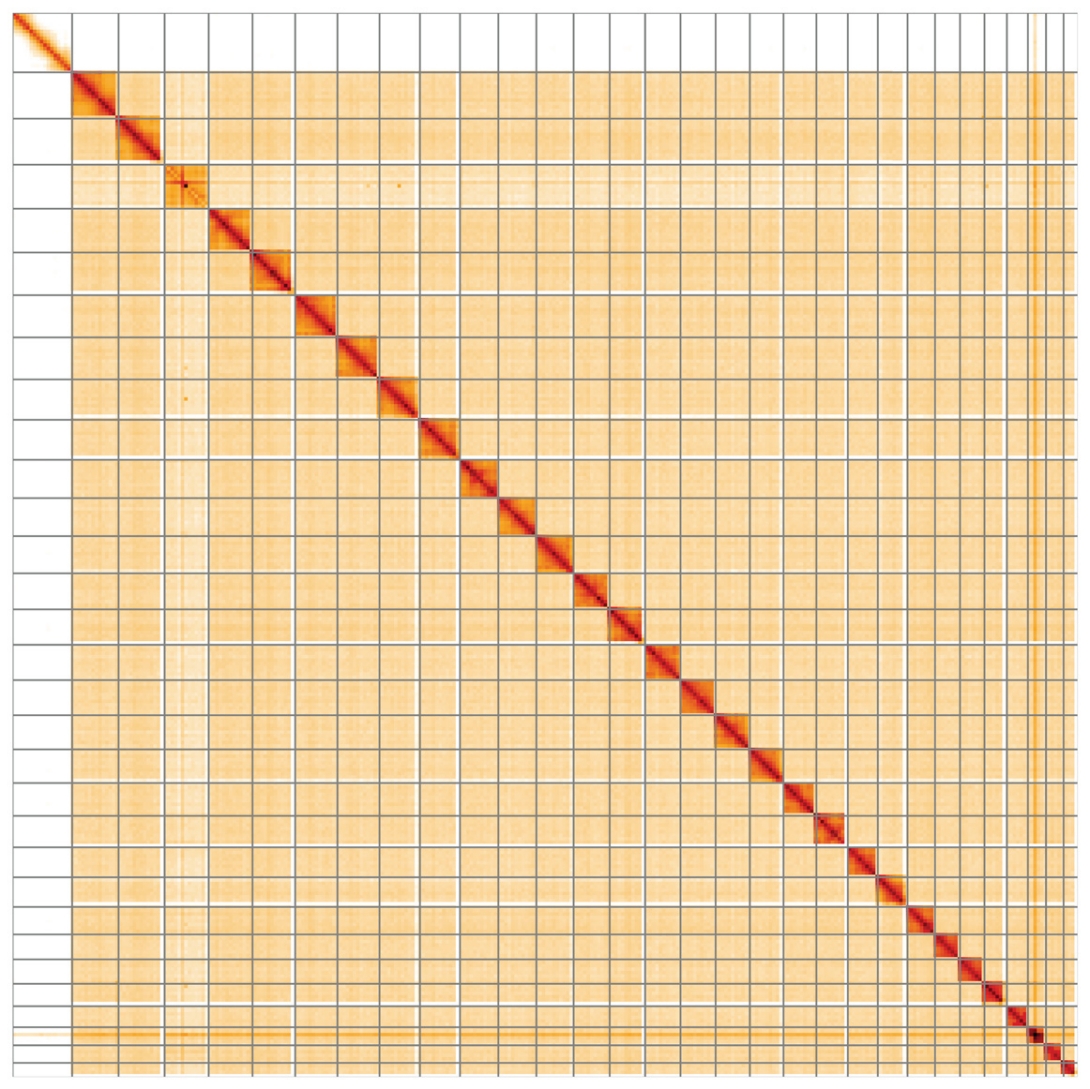
Genome assembly of *Nymphula nitidulata*, ilNymNiti1.1: Hi-C contact map of the ilNymNiti1.1 assembly, visualised using HiGlass. Chromosomes are shown in order of size from left to right and top to bottom. An interactive version of this figure may be viewed at https://genome-note-higlass.tol.sanger.ac.uk/l/?d=LBpzVD6nQHKGvY3czdx4Sg.

The final assembly has a total length of 635.8 Mb in 40 sequence scaffolds with a scaffold N50 of 22.2 Mb ( Table 1). The snail plot in Figure 2 provides a summary of the assembly statistics, while the distribution of assembly scaffolds on GC proportion and coverage is shown in Figure 3. The cumulative assembly plot in Figure 4 shows curves for subsets of scaffolds assigned to different phyla. Most (99.89%) of the assembly sequence was assigned to 31 chromosomal-level scaffolds, representing 30 autosomes and the Z and W sex chromosomes. Chromosome-scale scaffolds confirmed by the Hi-C data are named in order of size ( Figure 5; Table 2). While not fully phased, the assembly deposited is of one haplotype. Contigs corresponding to the second haplotype have also been deposited. The mitochondrial genome was also assembled and can be found as a contig within the multifasta file of the genome submission.

**Table 1.** Genome data for *Nymphula nitidulata*, ilNymNiti1.1.

Project accession data		
Assembly identifier	ilNymNiti1.1	
Species	*Nymphula nitidulata*	
Specimen	ilNymNiti1	
NCBI taxonomy ID	1594316	
BioProject	PRJEB55337	
BioSample ID	SAMEA7701288	
Isolate information	ilNymNiti1, female: whole organism (DNA sequencing) ilNymNiti2: whole organism (Hi-C sequencing)	
Assembly metrics *		*Benchmark*
Consensus quality (QV)	66.7	*≥ 50*
*k*-mer completeness	100.0%	*≥ 95%*
BUSCO **	C:98.9%[S:98.6%,D:0.3%], F:0.2%,M:0.9%,n:5,286	*C ≥ 95%*
Percentage of assembly mapped to chromosomes	99.89%	*≥ 95%*
Sex chromosomes	ZW	*localised homologous pairs*
Organelles	Mitochondrial genome: 15.36 kb	*complete single alleles*
Raw data accessions		
PacificBiosciences SEQUEL II	ERR10077560, ERR10077561	
Hi-C Illumina	ERR10084068	
Genome assembly		
Assembly accession	GCA_947347705.1	
*Accession of alternate haplotype*	GCA_947347715.1	
Span (Mb)	635.8	
Number of contigs	46	
Contig N50 length (Mb)	21.2	
Number of scaffolds	40	
Scaffold N50 length (Mb)	22.2	
Longest scaffold (Mb)	35.42	

* Assembly metric benchmarks are adapted from column VGP-2020 of “Table 1: Proposed standards and metrics for defining genome assembly quality” from ( Rhie *et al.*, 2021). ** BUSCO scores based on the lepidoptera_odb10 BUSCO set using version 5.3.2. C = complete [S = single copy, D = duplicated], F = fragmented, M = missing, n = number of orthologues in comparison. A full set of BUSCO scores is available at https://blobtoolkit.genomehubs.org/view/CANAFG01/dataset/CANAFG01/busco.

**Table 2.** Chromosomal pseudomolecules in the genome assembly of Nymphula nitidulata, ilNymNiti1.

INSDC accession	Chromosome	Length (Mb)	GC%
OX374617.1	1	27.66	36.5
OX374618.1	2	27.49	36.5
OX374620.1	3	26.07	36.5
OX374621.1	4	25.45	36.5
OX374622.1	5	25.21	36.0
OX374623.1	6	25.12	36.5
OX374624.1	7	24.02	36.5
OX374625.1	8	23.85	36.5
OX374626.1	9	22.88	36.5
OX374627.1	10	22.68	36.5
OX374628.1	11	22.21	36.5
OX374629.1	12	21.46	36.0
OX374630.1	13	21.17	36.5
OX374631.1	14	21.06	36.5
OX374632.1	15	20.94	36.5
OX374633.1	16	20.38	36.5
OX374634.1	17	20.04	37.0
OX374635.1	18	19.62	36.5
OX374636.1	19	18.71	37.0
OX374637.1	20	17.9	36.5
OX374638.1	21	17.71	37.0
OX374639.1	22	16.36	37.0
OX374640.1	23	14.9	37.0
OX374641.1	24	14.63	37.0
OX374642.1	25	13.29	37.5
OX374643.1	26	12.54	37.0
OX374644.1	27	10.78	38.5
OX374645.1	28	10.45	38.5
OX374646.1	29	8.81	37.5
OX374619.1	W	26.28	38.0
OX374616.1	Z	35.42	36.0
OX374647.1	MT	0.02	20.5

The estimated Quality Value (QV) of the final assembly is 66.7 with *k*-mer completeness of 100.0%, and the assembly has a BUSCO v5.3.2 completeness of 98.9% (single = 98.6%, duplicated = 0.3%), using the lepidoptera_odb10 reference set ( *n* = 5,286).

Metadata for specimens, barcode results, spectra estimates, sequencing runs, contaminants and pre-curation assembly statistics are given at https://links.tol.sanger.ac.uk/species/1594316.

## Genome annotation report

The *Nymphula nitidulata* genome assembly (GCA_947347705.1) was annotated using the Ensembl rapid annotation pipeline at the European Bioinformatics Institute (EBI). The resulting annotation includes 20,208 transcribed mRNAs from 20,031 protein-coding genes ( Table 1; https://rapid.ensembl.org/Nymphula_nitidulata_GCA_947347705.1/Info/Index).

## Methods

### Sample acquisition and nucleic acid extraction

The specimens of *Nymphula nitidulata* used for genome sequencing (specimen ID Ox000517, ToLID ilNymNiti1) and Hi-C sequencing (specimen ID Ox000518, ToLID ilNymNiti2) were collected from Wytham Woods, Oxfordshire (biological vice-county Berkshire), UK (latitude 51.77, longitude -1.33) on 2020-06-25 using a light trap. The specimens were collected and identified by Douglas Boyes (University of Oxford) and preserved on dry ice.

Protocols developed by the Wellcome Sanger Institute (WSI) Tree of Life core laboratory have been deposited on protocols.io ( Denton *et al.*, 2023b). The workflow for high molecular weight (HMW) DNA extraction at the WSI includes a sequence of core procedures: sample preparation; sample homogenisation, DNA extraction, fragmentation, and clean-up. In sample preparation, the ilNymNiti1 sample was weighed and dissected on dry ice ( Jay *et al.*, 2023). Whole organism tissue was homogenised using a PowerMasher II tissue disruptor ( Denton *et al.*, 2023a). HMW DNA was extracted using the Automated MagAttract v1 protocol ( Sheerin *et al.*, 2023). HMW DNA was sheared into an average fragment size of 12–20 kb in a Megaruptor 3 system with speed setting 30 ( Todorovic *et al.*, 2023). Sheared DNA was purified by solid-phase reversible immobilisation ( Strickland *et al.*, 2023): in brief, the method employs a 1.8X ratio of AMPure PB beads to sample to eliminate shorter fragments and concentrate the DNA. The concentration of the sheared and purified DNA was assessed using a Nanodrop spectrophotometer and Qubit Fluorometer and Qubit dsDNA High Sensitivity Assay kit. Fragment size distribution was evaluated by running the sample on the FemtoPulse system.

### Sequencing

Pacific Biosciences HiFi circular consensus DNA sequencing libraries were constructed according to the manufacturers’ instructions. DNA sequencing was performed by the Scientific Operations core at the WSI on a Pacific Biosciences SEQUEL II (HiFi) instruments. Hi-C data were also generated from whole organism tissue of ilNymNiti2 using the Arima2 kit and sequenced on the Illumina NovaSeq 6000 instrument.

### Genome assembly, curation and evaluation

Assembly was carried out with Hifiasm ( Cheng *et al.*, 2021) and haplotypic duplication was identified and removed with purge_dups ( Guan *et al.*, 2020). The assembly was then scaffolded with Hi-C data ( Rao *et al.*, 2014) using YaHS ( Zhou *et al.*, 2023). The assembly was checked for contamination and corrected as described previously ( Howe *et al.*, 2021). Manual curation was performed using HiGlass ( Kerpedjiev *et al.*, 2018) and PretextView ( Harry, 2022). The mitochondrial genome was assembled using MitoHiFi ( Uliano-Silva *et al.*, 2023), which runs MitoFinder ( Allio *et al.*, 2020) or MITOS ( Bernt *et al.*, 2013) and uses these annotations to select the final mitochondrial contig and to ensure the general quality of the sequence.

A Hi-C map for the final assembly was produced using bwa-mem2 ( Vasimuddin *et al.*, 2019) in the Cooler file format ( Abdennur & Mirny, 2020). To assess the assembly metrics, the *k*-mer completeness and QV consensus quality values were calculated in Merqury ( Rhie *et al.*, 2020). This work was done using Nextflow ( Di Tommaso *et al.*, 2017) DSL2 pipelines “sanger-tol/readmapping” ( Surana *et al.*, 2023a) and “sanger-tol/genomenote” ( Surana *et al.*, 2023b). The genome was analysed within the BlobToolKit environment ( Challis *et al.*, 2020) and BUSCO scores ( Manni *et al.*, 2021; Simão *et al.*, 2015) were calculated.

Table 3 contains a list of relevant software tool versions and sources.

**Table 3.** Software tools: versions and sources

Software tool	Version	Source
BlobToolKit	4.0.7	https://github.com/blobtoolkit/blobtoolkit
BUSCO	5.3.2	https://gitlab.com/ezlab/busco
Hifiasm	0.16.1-r375	https://github.com/chhylp123/hifiasm
HiGlass	1.11.6	https://github.com/higlass/higlass
Merqury	MerquryFK	https://github.com/thegenemyers/MERQURY.FK
MitoHiFi	2	https://github.com/marcelauliano/MitoHiFi
PretextView	0.2	https://github.com/wtsi-hpag/PretextView
purge_dups	1.2.3	https://github.com/dfguan/purge_dups
sanger-tol/genomenote	v1.0	https://github.com/sanger-tol/genomenote
sanger-tol/readmapping	1.1.0	https://github.com/sanger-tol/readmapping/tree/1.1.0
YaHS	yahs-1.1.91eebc2	https://github.com/c-zhou/yahs

### Genome annotation

The BRAKER2 pipeline ( Brůna *et al.*, 2021) was used in the default protein mode to generate annotation for the *Nymphula nitidulata* assembly (GCA_947347705.1) in Ensembl Rapid Release at the EBI.

### Wellcome Sanger Institute – Legal and Governance

The materials that have contributed to this genome note have been supplied by a Darwin Tree of Life Partner. The submission of materials by a Darwin Tree of Life Partner is subject to the **‘Darwin Tree of Life Project Sampling Code of Practice’**, which can be found in full on the Darwin Tree of Life website here. By agreeing with and signing up to the Sampling Code of Practice, the Darwin Tree of Life Partner agrees they will meet the legal and ethical requirements and standards set out within this document in respect of all samples acquired for, and supplied to, the Darwin Tree of Life Project.

Further, the Wellcome Sanger Institute employs a process whereby due diligence is carried out proportionate to the nature of the materials themselves, and the circumstances under which they have been/are to be collected and provided for use. The purpose of this is to address and mitigate any potential legal and/or ethical implications of receipt and use of the materials as part of the research project, and to ensure that in doing so we align with best practice wherever possible. The overarching areas of consideration are:

•     Ethical review of provenance and sourcing of the material

•     Legality of collection, transfer and use (national and international)

Each transfer of samples is further undertaken according to a Research Collaboration Agreement or Material Transfer Agreement entered into by the Darwin Tree of Life Partner, Genome Research Limited (operating as the Wellcome Sanger Institute), and in some circumstances other Darwin Tree of Life collaborators.

## Data Availability

European Nucleotide Archive: *Nymphula nitidulata*. Accession number PRJEB55337; https://identifiers.org/ena.embl/PRJEB55337 ( Wellcome Sanger Institute, 2022). The genome sequence is released openly for reuse. The *Nymphula nitidulata* genome sequencing initiative is part of the Darwin Tree of Life (DToL) project. All raw sequence data and the assembly have been deposited in INSDC databases. Raw data and assembly accession identifiers are reported in Table 1.

## Author information

Members of the University of Oxford and Wytham Woods Genome Acquisition Lab are listed here: https://doi.org/10.5281/zenodo.7125292.

Members of the Darwin Tree of Life Barcoding collective are listed here: https://doi.org/10.5281/zenodo.4893703.

Members of the Wellcome Sanger Institute Tree of Life Management, Samples and Laboratory team are listed here: https://doi.org/10.5281/zenodo.10066175.

Members of Wellcome Sanger Institute Scientific Operations: Sequencing Operations are listed here: https://doi.org/10.5281/zenodo.10043364.

Members of the Wellcome Sanger Institute Tree of Life Core Informatics team are listed here: https://doi.org/10.5281/zenodo.10066637.

Members of the Tree of Life Core Informatics collective are listed here: https://doi.org/10.5281/zenodo.5013541.

Members of the Darwin Tree of Life Consortium are listed here: https://doi.org/10.5281/zenodo.4783558.

## References

[ref-1] Abdennur N Mirny LA : Cooler: Scalable storage for Hi-C data and other genomically labeled arrays. *Bioinformatics.* 2020;36(1):311–316. 10.1093/bioinformatics/btz540 31290943 PMC8205516

[ref-2] Allio R Schomaker-Bastos A Romiguier J : MitoFinder: Efficient automated large-scale extraction of mitogenomic data in target enrichment phylogenomics. *Mol Ecol Resour.* 2020;20(4):892–905. 10.1111/1755-0998.13160 32243090 PMC7497042

[ref-3] Bernt M Donath A Jühling F : MITOS: Improved *de novo* metazoan mitochondrial genome annotation. *Mol Phylogenet Evol.* 2013;69(2):313–319. 10.1016/j.ympev.2012.08.023 22982435

[ref-31] Brůna T Hoff KJ Lomsadze A : BRAKER2: Automatic eukaryotic genome annotation with GeneMark-EP+ and AUGUSTUS supported by a protein database. *NAR Genom Bioinform.* 2021;3(1): lqaa108. 10.1093/nargab/lqaa108 33575650 PMC7787252

[ref-4] Challis R Richards E Rajan J : BlobToolKit – interactive quality assessment of genome assemblies. *G3 (Bethesda).* 2020;10(4):1361–1374. 10.1534/g3.119.400908 32071071 PMC7144090

[ref-5] Cheng H Concepcion GT Feng X : Haplotype-resolved *de novo* assembly using phased assembly graphs with hifiasm. *Nat Methods.* 2021;18(2):170–175. 10.1038/s41592-020-01056-5 33526886 PMC7961889

[ref-6] De-Freitas I De Agostini J Stefani V : The Aquatic Lepidopterans: A Mysterious and Unknown Fauna. In: Del-Claro, K. and Guillermo, R. (eds.) *Aquatic Insects*. Cham: Springer International Publishing,2019;341–347. 10.1007/978-3-030-16327-3_13

[ref-7] Denton A Oatley G Cornwell C : Sanger Tree of Life Sample Homogenisation: PowerMash. *protocols.io.* 2023a. 10.17504/protocols.io.5qpvo3r19v4o/v1

[ref-8] Denton A Yatsenko H Jay J : Sanger Tree of Life Wet Laboratory Protocol Collection V.1. *protocols.io.* 2023b. 10.17504/protocols.io.8epv5xxy6g1b/v1

[ref-9] Di Tommaso P Chatzou M Floden EW : Nextflow enables reproducible computational workflows. *Nat Biotechnol.* 2017;35(4):316–319. 10.1038/nbt.3820 28398311

[ref-10] Guan D McCarthy SA Wood J : Identifying and removing haplotypic duplication in primary genome assemblies. *Bioinformatics.* 2020;36(9):2896–2898. 10.1093/bioinformatics/btaa025 31971576 PMC7203741

[ref-11] Harry E : PretextView (Paired REad TEXTure Viewer): A desktop application for viewing pretext contact maps. 2022; [Accessed 19 October 2022]. Reference Source

[ref-12] Hotaling S Kelley JL Frandsen PB : Aquatic Insects Are Dramatically Underrepresented in Genomic Research. *Insects.* 2020;11(9):601. 10.3390/insects11090601 32899516 PMC7563230

[ref-13] Howe K Chow W Collins J : Significantly improving the quality of genome assemblies through curation. *GigaScience.* 2021;10(1): giaa153. 10.1093/gigascience/giaa153 33420778 PMC7794651

[ref-14] Jay J Yatsenko H Narváez-Gómez JP : Sanger Tree of Life Sample Preparation: Triage and Dissection. *protocols.io.* 2023. 10.17504/protocols.io.x54v9prmqg3e/v1

[ref-15] Kerpedjiev P Abdennur N Lekschas F : HiGlass: web-based visual exploration and analysis of genome interaction maps. *Genome Biol.* 2018;19(1): 125. 10.1186/s13059-018-1486-1 30143029 PMC6109259

[ref-16] Manni M Berkeley MR Seppey M : BUSCO update: Novel and streamlined workflows along with broader and deeper phylogenetic coverage for scoring of eukaryotic, prokaryotic, and viral genomes. *Mol Biol Evol.* 2021;38(10):4647–4654. 10.1093/molbev/msab199 34320186 PMC8476166

[ref-17] Pabis K : What is a moth doing under water? Ecology of aquatic and semi-aquatic Lepidoptera. *Knowl Manag Aquat Ecosyst.* 2018; (419): 42. 10.1051/kmae/2018030

[ref-18] Rao SSP Huntley MH Durand NC : A 3D map of the human genome at kilobase resolution reveals principles of chromatin looping. *Cell.* 2014;159(7):1665–1680. 10.1016/j.cell.2014.11.021 25497547 PMC5635824

[ref-19] Rhie A McCarthy SA Fedrigo O : Towards complete and error-free genome assemblies of all vertebrate species. *Nature.* 2021;592(7856):737–746. 10.1038/s41586-021-03451-0 33911273 PMC8081667

[ref-20] Rhie A Walenz BP Koren S : Merqury: Reference-free quality, completeness, and phasing assessment for genome assemblies. *Genome Biol.* 2020;21(1): 245. 10.1186/s13059-020-02134-9 32928274 PMC7488777

[ref-21] Sheerin E Sampaio F Oatley G : Sanger Tree of Life HMW DNA Extraction: Automated MagAttract v.1. *protocols.io.* 2023. 10.17504/protocols.io.x54v9p2z1g3e/v1

[ref-22] Simão FA Waterhouse RM Ioannidis P : BUSCO: assessing genome assembly and annotation completeness with single-copy orthologs. *Bioinformatics.* 2015;31(19):3210–3212. 10.1093/bioinformatics/btv351 26059717

[ref-23] Strickland M Cornwell C Howard C : Sanger Tree of Life Fragmented DNA clean up: Manual SPRI. *Protocols.Io.* 2023. 10.17504/protocols.io.kxygx3y1dg8j/v1

[ref-24] Surana P Muffato M Qi G : sanger-tol/readmapping: sanger-tol/readmapping v1.1.0 - Hebridean Black (1.1.0). *Zenodo.* 2023a. 10.5281/zenodo.7755669

[ref-25] Surana P Muffato M Sadasivan Baby C : sanger-tol/genomenote (v1.0.dev). *Zenodo.* 2023b. 10.5281/zenodo.6785935

[ref-26] Todorovic M Sampaio F Howard C : Sanger Tree of Life HMW DNA Fragmentation: Diagenode Megaruptor®3 for PacBio HiFi. *protocols.io.* 2023. 10.17504/protocols.io.8epv5x2zjg1b/v1

[ref-27] Uliano-Silva M Ferreira JGRN Krasheninnikova K : MitoHiFi: a python pipeline for mitochondrial genome assembly from PacBio high fidelity reads. *BMC Bioinformatics.* 2023;24(1): 288. 10.1186/s12859-023-05385-y 37464285 PMC10354987

[ref-28] Vasimuddin M Misra S Li H : Efficient Architecture-Aware Acceleration of BWA-MEM for Multicore Systems.In: *2019 IEEE International Parallel and Distributed Processing Symposium (IPDPS).*IEEE,2019;314–324. 10.1109/IPDPS.2019.00041

[ref-30] Wellcome Sanger Institute: The genome sequence of the Beautiful China-mark moth *Nymphula nitidulata* (Hufnagel, 1767). European Nucleotide Archive. [dataset], accession number PRJEB55337,2022.

[ref-29] Zhou C McCarthy SA Durbin R : YaHS: yet another Hi-C scaffolding tool. *Bioinformatics.* 2023;39(1): btac808. 10.1093/bioinformatics/btac808 36525368 PMC9848053

