# Supplementary material for: The genome sequence of the Beautiful China-mark moth Nymphula nitidulata (Hufnagel, 1767)
Source: Wellcome Open Res. 2024 Mar 8;9:135. [Version 1] doi: 10.12688/wellcomeopenres.21102.1 (PMC11301135; doi:10.12688/wellcomeopenres.21102.1)
Supplement: Peer review history

# Reviewer report: Reviewer response for version 1

**DOI:** 10.21956/wellcomeopenres.23342.r89795

**Published:** 2024-08-05

**Authors:** Melody Clark

**Affiliations:** 1 Natural Environment Research Council, Cambridge, UK

This data note summarises the genome assembly from an individual female *Nymphula nitidulata* (the Beautiful China-mark moth). The case is well-made for sequencing the genome of this species. This individual was captured in Wytham Woods, one of the most intensively researched areas of woodland in the world. Thus, these genome data contribute, not only to the Darwin Tree of Life dataset, but are also associated with one of the UKs most significant long-term ecological surveys. The sequencing of a member of this particular sub-family will be particularly useful for understanding adaptation to a freshwater habitat.

 The DNA for the genome sequence was extracted using standard Darwin Tree of Life protocols and also the sequencing and sequencing pipeline were also standard for the Darwin Tree of Life. Hence, the assembly is of very high quality, with a BUSCO completeness score of 98.9%. The sequences are scaffolded into 31 chromosomal pseudomolecules, including the sex chromosomes. This will be a very useful resource for future studies in a variety of different areas: evolution, ecological adaptation, sex chromosomes and entomology.

Are sufficient details of methods and materials provided to allow replication by others?

Yes

Is the rationale for creating the dataset(s) clearly described?

Yes

Are the datasets clearly presented in a useable and accessible format?

Yes

Are the protocols appropriate and is the work technically sound?

Yes

Reviewer Expertise:

Genomics, transcriptomics, ecological adaptation

I confirm that I have read this submission and believe that I have an appropriate level of expertise to confirm that it is of an acceptable scientific standard.

# Reviewer report: Reviewer response for version 1

**DOI:** 10.21956/wellcomeopenres.23342.r88073

**Published:** 2024-07-09

**Authors:** Axel Künstner

**Affiliations:** 1 University of Lübeck, Lübeck, Germany

The authors present the genome assembly from an individual Beautiful China-mark moth ( *Nymphula nitidulata*).

 The manuscript is clearly written, methods well explained and I did not found any technical flaws in the assembly strategy. The raw and the assembled data is available via ENA.

 I do not have any comments to improve the presented manuscript.

Are sufficient details of methods and materials provided to allow replication by others?

Yes

Is the rationale for creating the dataset(s) clearly described?

Yes

Are the datasets clearly presented in a useable and accessible format?

Yes

Are the protocols appropriate and is the work technically sound?

Yes

Reviewer Expertise:

My area of research is mainly Bioinformatics.

I confirm that I have read this submission and believe that I have an appropriate level of expertise to confirm that it is of an acceptable scientific standard.
